# Adaptive Strategies in a Poly-Extreme Environment: Differentiation of Vegetative Cells in *Serratia ureilytica* and Resistance to Extreme Conditions

**DOI:** 10.3389/fmicb.2019.00102

**Published:** 2019-02-05

**Authors:** Sevasti Filippidou, Thomas Junier, Tina Wunderlin, Wafa M. Kooli, Ilona Palmieri, Andrej Al-Dourobi, Veronica Molina, Reto Lienhard, Jorge E. Spangenberg, Shannon L. Johnson, Patrick S. G. Chain, Cristina Dorador, Pilar Junier

**Affiliations:** ^1^Laboratory of Microbiology, University of Neuchatel, Neuchatel, Switzerland; ^2^Microbial Ecology Group, Centre for Ecology and Hydrology, Wallingford, United Kingdom; ^3^Space Microbiology Research Group, Radiation Biology Department, Institute of Aerospace Medicine, German Aerospace Center (DLR e.V.), Cologne, Germany; ^4^Vital-IT Group, Swiss Institute of Bioinformatics, Lausanne, Switzerland; ^5^Departamento de Biología, Facultad de Ciencias Naturales y Exactas, Universidad de Playa Ancha, Valparaíso, Chile; ^6^ADMED Microbiologie, La Chaux-de-Fonds, Switzerland; ^7^Institute of Earth Surface Dynamics, University of Lausanne, Lausanne, Switzerland; ^8^Los Alamos National Laboratory, Los Alamos, NM, United States; ^9^Laboratorio de Complejidad Microbiana y Ecología Funcional, Departamento de Biotecnología, Facultad de Ciencias del Mar y Recursos Biológicos, Universidad de Antofagasta, Antofagasta, Chile; ^10^Centre for Biotechnology and Bioengineering, Antofagasta, Chile

**Keywords:** differentiation, dormancy, extreme environments, harsh conditions, *Serratia*, sporulation, resistance

## Abstract

Poly-extreme terrestrial habitats are often used as analogs to extra-terrestrial environments. Understanding the adaptive strategies allowing bacteria to thrive and survive under these conditions could help in our quest for extra-terrestrial planets suitable for life and understanding how life evolved in the harsh early earth conditions. A prime example of such a survival strategy is the modification of vegetative cells into resistant resting structures. These differentiated cells are often observed in response to harsh environmental conditions. The environmental strain (strain Lr5/4) belonging to *Serratia ureilytica* was isolated from a geothermal spring in Lirima, Atacama Desert, Chile. The Atacama Desert is the driest habitat on Earth and furthermore, due to its high altitude, it is exposed to an increased amount of UV radiation. The geothermal spring from which the strain was isolated is oligotrophic and the temperature of 54°C exceeds mesophilic conditions (15 to 45°C). Although the vegetative cells were tolerant to various environmental insults (desiccation, extreme pH, glycerol), a modified cell type was formed in response to nutrient deprivation, UV radiation and thermal shock. Scanning (SEM) and Transmission Electron Microscopy (TEM) analyses of vegetative cells and the modified cell structures were performed. In SEM, a change toward a circular shape with reduced size was observed. These circular cells possessed what appears as extra coating layers under TEM. The resistance of the modified cells was also investigated, they were resistant to wet heat, UV radiation and desiccation, while vegetative cells did not withstand any of those conditions. A phylogenomic analysis was undertaken to investigate the presence of known genes involved in dormancy in other bacterial clades. Genes related to spore-formation in *Myxococcus* and Firmicutes were found in *S. ureilytica* Lr5/4 genome; however, these genes were not enough for a full sporulation pathway that resembles either group. Although, the molecular pathway of cell differentiation in *S. ureilytica* Lr5/4 is not fully defined, the identified genes may contribute to the modified phenotype in the *Serratia* genus. Here, we show that a modified cell structure can occur as a response to extremity in a species that was previously not known to deploy this strategy. This strategy may be widely spread in bacteria, but only expressed under poly-extreme environmental conditions.

## Introduction

The Biospace corresponds to the physicochemical conditions under which life can be sustained (Cockell, 2015). On Earth, both mesophilic and extremophilic habitats are part of the Biospace, but only extreme habitats are considered as analogs to Young Earth or other planets and exoplanets, making them suitable sites to study the origin and evolution of life, as well as the extraterrestrial Biospace. Extreme habitats are considered challenging ecosystems for life because physicochemical parameters deviate from those used to describe mesophilic conditions sustaining life. However, on Earth, a diverse microbial community inhabit extreme ecosystems. This is supported, for example, by diversity surveys on terrestrial and oceanic geothermal sites (Schubotz et al., 2013), saline environments [salt lakes, seashore evaporations, or estuaries; (Edwards, 1990)], acidic and alkaline environments (Edwards, 1990; Sorokin et al., 2014) and ecosystems that are highly radiated (Sghaier et al., 2007; Cordero et al., 2018). Although based on a biased perception of extremity we tend to consider extreme habitats as more challenging for survival, fluctuations in environmental parameters even in non-extreme habitats, would lead to periods in which active growth is compromised (Sadoff, 1973).

In order to withstand adversity, life adapts by deploying various survival strategies, one of which is cellular differentiation into a more resistant form. In eukaryotes, cells containing the same genetic information can transform into a myriad of differentiated cell types [for instance in the infamous case of stem cell differentiation to a broad range of specialized cells (indicative reference: Engler et al., 2006)]. In contrast to the complexity and cellular diversity of eukaryotes, prokaryotic cells are perceived as possessing simpler morphologies. However, they do differentiate into various resistant forms, such as endospores (Firmicutes), myxospores (δ-Proteobacteria), akinetes (Cyanobacteria), and exospores (Actinobacteria) (Barton, 2005). These differentiated structures offer the advantage of resisting through adverse times under resting conditions (low metabolic activity), with the possibility to deploy full metabolic activity and cellular division once conditions become favorable.

Reports of dormancy involving specialized cellular structures outside these four groups are also found in literature. These include the proposed formation of endospores in the purple non-sulfur phototroph α-Proteobacterium *Rhodobacter johrii* JA192, which produced light-refracting structures visible under phase contrast microscopy and malachite green staining (Girija et al., 2010). Likewise, cyst formation has been shown in *Rhodospirillum centenum*, a photosynthetic member of the *Azospirillum* clade with a complex developmental life cycle (Dong and Bauer, 2015). Another example, which has been at the center of recent debate, was the production of phase-bright granules similar to endospores in Mycobacteria (Ghosh et al., 2009). Although data of mRNA expression for some key sporulation-related genes was provided in the case of Mycobacteria (Ghosh et al., 2009), subsequent genomic analysis revealed the absence of the core set of sporulation genes identified for Firmicutes and production of phase-bright structures was irreproducible by other researchers (Traag et al., 2010). This latter example, shows some of the challenges of describing sporulation in a clade previously considered as asporogenic. Formation of endospores has been also suggested for *Serratia* (Ajithkumar et al., 2003). The genus *Serratia* belongs to γ-Proteobacteria, to the family Enterobacteriaceae, and some of its members are of clinical importance (Grimont and Grimont, 2015). Production of concentric “spores” in response to heat-shock was reported in *Serratia marcescens* subsp. *sakuensis* (Ajithkumar et al., 2003). This strain was isolated from a wastewater treatment tank, alongside several strains belonging to the genus *Bacillus*, leading the authors to speculate at horizontal gene transfer to explain the origin of sporulation in *Serratia*. In spite of these reports, the formation of dormant cells outside the four thoroughly studied taxa have always been received with skepticism, and in particular in the case of endospore formation, which is so far the best studied example of a dormant cell in Bacteria. Endospores arise from asymmetrical cell division and a complex morphogenetic differentiation program (Way, 1996). Because of its complexity, endospore formation is supposed to be restricted to Firmicutes (Traag et al., 2010). In the case of other differentiated dormant cells such as exospores, myxospores or cysts, the mechanisms of formation are much less understood (Abel-Santos, 2012), limiting our ability to perform comparative studies in order to assess their prevalence.

Over the last three decades, the formation of cyst-like resting structures has been proposed for diverse non-spore-formers (Sadasivan and Neyra, 1985; Suzina et al., 2004). These cyst-like cells share the characteristics of refracting light in the phase contrast microscope, survival over long periods of time, and revival under optimal conditions (Muliukin et al., 1997). Although these reports expand our understanding of dormancy as a survival strategy in response to conditions limiting active growth, the current knowledge on environmental triggers for the production of these cyst-like resting cells is mainly limited to nutrient deprivation. Moreover, the resistance limits of these structures and the genetic background of this process are not yet studied.

In this study, we report the discovery of cyst-like cells in *S. ureilytica* str. Lr5/4, which was isolated in a geothermal spring in Lirima, Atacama Desert, Chile. We provide evidence that this cyst-like cell form is more resistant than the vegetative cell it derived from. The environmental factors triggering the formation of cyst-like cells, the resistance of these structures and their biochemical properties, as well as the genomic imprints that could be related to cyst production are studied herein. Moreover, whether the phenomenon of resting cells in non-spore forming bacteria is widespread remains unanswered. In order to address this question, phylogenomic and functional analysis of genes related to resting structures was performed. Overall, with this work we attempt to shed light on the survival behavior of cyst-forming mesophilic bacteria in extreme environments and discuss the ecological relevance of these bacteria.

## Materials and Methods

### Sampling and Isolation

**Figure 1:**
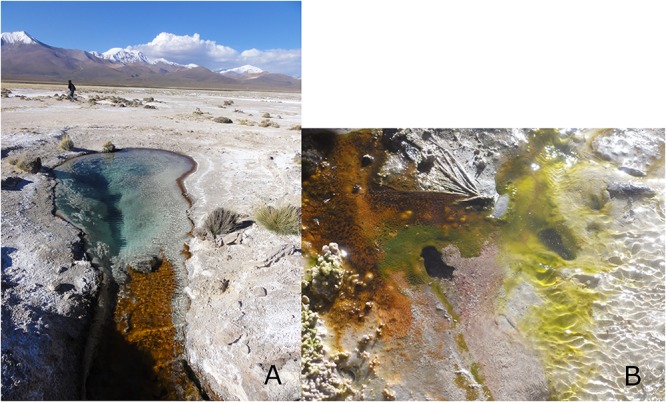
**(A)** The sampling site of the geothermal pond in Lirima, Chile, from which *S. ureilytica* Lr5/4 was isolated. **(B)** A close-up photograph of the microbial mat.

Lirima is a geothermal site located in the Andean highlands of northern Chile (3.997 m.a.s.l., 19°51.118W, 68°54.402S). The Chilean Altiplano presents multiple co-occurring environmental stressors: the highest UV radiation ever reported on Earth (Cordero et al., 2016); variable daily temperatures (from below 0°C up to 30°C); high altitude (4000 m); and low relative humidity (Cordero et al., 2016). Microbial mats in different geothermal environments (aqueous and non-aqueous, temperature ranging from 30°C to over 90°C) were sampled during a field campaign in April 2011. The microbial mats were collected and stored using sterile material. After sampling, the sample was preserved at 4°C until enrichment. For aerobic enrichment, one gram of homogenized mat was inoculated in nutrient broth (NB) (Biolife, Italy), pH adjusted to 7.2, and incubated at room temperature for 7 days under aerobic conditions. The mismatch between *in situ* and cultivation temperature was used to favor the selection of dormant forms. 100 μL of the enrichment culture was plated on nutrient agar (NA) (Biolife, Italy) and incubated for 24 h at room temperature (RT) under aerobic conditions. Axenic cultures were isolated after repeated serial dilutions on NA medium at RT for 24 h. Strains were confirmed for purity by microscopic observation. Pure isolates were cryopreserved in 30% (v/v) of glycerol at -80°C. Isolates were screened for phase bright spore-like structures by microscopy after having been selected for dormancy by nutrient deprivation. The isolate Lr5/4 described here was isolated from a double layer microbial mat in a source pond at 54°C (Figure 1), alongside four other strains that were identified as belonging to the genus *Bacillus*.

### Physiological Characterization of Lr5/4

Cell growth was monitored at different temperatures (4, 15, 25, 30, 37, 45, 55, and 60°C) for up to 72 h in nutrient broth medium (Biolife, Italy), measuring optical density at 600 nm (OD600) with a spectrophotometer Genesys 10S UV-Vis Thermo Scientific (Thermo Fisher Scientific Inc., United States). In addition, growth was measured at different pH (2 to 13) and at different NaCl concentrations (1, 2, 3, 3.5, 4, 5, 5.5, 6, 6.5, 8, 9, 10, 11, and 12% w/v) with a microplate reader at 590 nm (UVM 340 Microplate Reader, ASYS, Hiteck, United Kingdom) for up to 72 h. The need for oxygen during growth was verified using the method of thioglycollate medium (Brewer, 1940).

Phospholipids were extracted from the whole cells using a single-phase (methanol/dichloromethane/phosphate buffer; 2:1:0.8; v/v/v) extraction procedure (Guckert et al., 1985). Fatty acid methyl esters (FAMES) were prepared from the extracted acyl lipid mixture by acid-catalyzed methanolysis (Ichihara and Fukubayashi, 2010). The chemical characterization of the lipids was performed with the Agilent gas chromatograph 6890 coupled to the Agilent 5973 quadrupole mass selective detector (GC-MS; Palo Alto, CA, United States). For the analysis of FAME fractions, the system was equipped with the Agilent free fatty acids phase (FFAP) fused silica capillary column (50 m length, 0.20 mm i.d.) coated with nitroterephthalic acid modified polyethylene glycol stationary phase (film thickness 0.33 μm). A sample aliquot was injected split-less at a temperature of 200°C. Helium was used as carrier gas (1 mL/min flow rate). After an initial period of 2 min at 100°C, the column was heated to 240°C at 5°C/min followed by an isothermal period of 30 min. The MS was operated in the electron impact mode at 70 eV, source temperature of 250°C, emission current of 1 mA and multiple-ion detection with a mass range from 50 to 600 amu. Compound identifications were based on comparison of standards, GC retention time, and mass spectrometric fragmentation patterns.

### Molecular Characterization

For G+C content analysis and DNA-DNA hybridization, the services of DSMZ, Germany were used. Strain Lr5/4 was tested against *S. marcescens* (DSMZ 30121) and *S. ureilytica* (DSMZ 16952). MALDI-TOF mass spectra were acquired on a Bruker Microflex RLF mass spectrometer (Bruker Daltonics, Bremen, Germany). Spectral mass resolutions and signal-to-noise ratios were determined with the software Flex Analysis 3.3.65 (Bruker Daltonics).

For PCR amplifications, genomic DNA was extracted using the InnuPREP Bacteria DNA Kit (Analytik Jena, Germany), according to the manufacturer’s instructions. DNA was quantified fluorometrically with a Qubit^®^ dsDNA HS Assay Kit with a Qubit^®^ 2.0 Fluorometer (Invitrogen Ltd., Paisley, United Kingdom) according to the manufacturer’s instructions. PCR amplification of the 16S rRNA gene was performed using the primer set GM3F and GM4R (Muyzer et al., 1995). The PCR products were purified with a MultiScreen PCRμ96 Filter Plate (Millipore), according to the manufacturer’s instructions and sequenced using the services of GATC Biotech (Germany). To obtain the full-16S rRNA gene sequence, Lr5/4 PCR products were sequenced in addition with the primers 907r, 520r, and 926f primers (Muyzer et al., 1995; Forney et al., 1997). The 16S rRNA gene sequence was compared in GenBank by using the BLASTn tool (Altschul et al., 1997). After alignment, a series of sequences that had an identity match over 98% were selected, along with 16S rRNA gene sequences from cultured representative strains belonging to different species of the genus *Serratia* and a 16S rRNA gene sequence of a *Geobacillus* sp. (serving as an outgroup in order to root the tree). These sequences were used to build-up a cladogram using the online tools of phylogeny.fr website (Dereeper et al., 2008). Multiple sequence alignment has been performed using MUSCLE and the phylogenetic tree has been constructed using PhyML. To determine the confidence values for individual branches, 100 bootstrap replications were calculated for each generated tree.

### Morphological Characterization

Colonies of strain Lr5/4 were obtained and observed after overnight growth on NA. Gram staining was performed on an overnight solid culture using the Hucker staining method (Gerhardt, 1994). Spores were observed with a phase contrast microscope (Leica DM R, magnification 1000×). Vegetative cells and spores were also observed by Scanning and Transmission Electron Microscopy (SEM and TEM). Vegetative cells were observed from a fresh 24 h culture inoculated in NB. Cyst-like structures were collected from a 3-month old solid culture. Both preparations were fixed in 2.5% glutaraldehyde in a cacodylate buffer (0.1 M; pH7.4) for 2 h at room temperature and then overnight at 4°C. They were then washed by gentle immersion in cacodylate buffer (0.2M; pH7.4) post fixed with 1% OsO_4_ in the same buffer, and carefully washed with the above buffer. For SEM, the samples were dehydrated in 15 to 100% of ethanol solution and finally fixed on Poly-L-Lysine slides and coated with a 23 nm gold layer in a BaltecSCD005 sputter apparatus. The samples were observed with a Philips XL30 SEM at acceleration voltages of 10–20 kV. For TEM, the samples were dehydrated in 15–100% acetone and embedded in Spurr’s resin. Serial sections were made with a Reichert Ultracut-S microtome, mounted on copper grids, double stained with uranyl acetate and lead citrate, and observed with a Philips CM 100 TEM at 60 or 80 kV.

### Dormant Structure Production and Resistance Assays

Nutrient deprivation on solid media was one method for the production of light-refracting spherical cysts. Other environmental triggers were also put to test. Overnight liquid cultures in NB were subjected to temperature shocks. These shocks included two rounds of placing the liquid culture in an 80°C water bath for 20 min, then moved to 4°C for 20 min. The culture was then placed at room temperature overnight and observations for light refracting spheres was observed at 24 or 48 h. Overnight solid cultures on NA were subjected to desiccation and UV radiation, in order to verify whether these factors trigger the formation of cyst-like bodies. Overnight solid cultures were placed in a desiccator for 72 h until the solid medium dried to a 1 mm thin layer. Formation of light refracting spheres was examined at 24 and 48 h. Overnight solid cultures were also placed 60 cm from an UVc lamp (approximate wavelength 254 nm). The experiment was held under sterile conditions (BSL II hood), and the lids of the petri dishes were removed so that the bacterial colonies were directly exposed to the radiation. At *t* = 5, 10, 20, 30, 60, 120, 180, 240, and 720 min, three petri dishes were removed, closed sterilely and kept at room temperature. Observations for light refracting spheres was made at 24 or 48 h. Finally, overnight liquid cultures in NB were autoclaved and subjected to temperature extremes (no heat-shock). The extreme temperatures tested were -80, -20, 4, 60, 80, and 100°C. The overnight liquid cultures were incubated for 24 h at these temperatures, then observed for cyst formation. *S. ureilytica* str. Lr5/4 was tested along with *Bacillus subtilis* str. NEU1294, *Anoxybacillus* sp. str. Lr10/3 (isolated along Lr5/4 from Lirima), *S. marcescens* DSMZ 30121, *S. ureilytica* DSMZ 16952, and *Escherichia coli* str. NEU1006.

Spores produced by nutrient deprivation and heat-shocks were subjected to resistance tests. A heat resistance test for the spores of strain Lr5/4 was performed as previously described (Ajithkumar et al., 2003), along with *S. marcescens* (DSMZ 30121), *S. ureilytica* (DSMZ 16952), *Bacillus subtilis* (Neu1294), and *Anoxybacillus* sp. (Lr10/3). Suspensions of these cultures in tryptic soy broth (TSB) medium were heat-shocked at 70 and 75°C for 20 min and then re-cultured under optimal conditions. Growth was macroscopically verified after 24 and 48 h. Cyst-like or spore preparations, as well as vegetative cells from old *S. marcescens* str. DSMZ 30121, *S. ureilytica* str. DSMZ 16952, and *E. coli* str. NEU1006, were also autoclaved and exposed to UV radiation (radiation applied at 60 cm from a UV-C lamp, 200–280 nm) and to temperature extremes (as described above for cyst production). After exposure, biomass from the solid or liquid medium was stroked on solid media and cultured under optimal conditions in order to verify re-growth.

### DPA Measurement

The presence of dipicolinic acid (DPA) in the spores was assessed on a 3-month old Lr5/4 preparation (20 mg wet weight), according to a previously published method (Brandes Ammann et al., 2011). This preparation contained almost exclusively refracting light structures. Fluorescence was measured within a Perkin-Elmer LS50B fluorometer. The excitation wavelength was set at 272 nm with a slit width of 2.5 nm. Emission was measured at 545 nm (slit width 2.5 nm). The device was set in the phosphorescence mode (equivalent to time-resolved fluorescence). The delay between emission and measurement was set at 50 μs. Measurements were performed every 20 ms. The integration of signal was performed over a duration of 1.2 ms. Values recovered for each measurement corresponded to the mean of the relative fluorescence unit (RFU) values given by the instrument within the 30 s following sample introduction in the device. Finally, to transform RFU units into DPA concentrations, a 10-point standard curve was established using increasing concentrations of DPA from 0.5 up to 10 μM.

### Genome Sequencing

Genomic DNA was extracted from an overnight culture using the Genomic-tip 20/G Kit (Qiagen GmbH, Germany). Sequencing was performed with PacBio RS II system based on single molecule, real-time (SMRT) technology (Pacific Biosciences, California). Genome assembly was performed using hgap 2.0 in the SmrtPortal. The full closed genome of *S. ureilytica* strain Lr5/4 presents a unique contig of 5,39 Mbp in size, and a G+C content of 59.2%. Genome annotation utilized an Ergatis based (Hemmerich et al., 2010) workflow with minor manual curation. The circular genome with the additional features was created using DNAplotter (Carver et al., 2009). The genome sequence was submitted to NCBI under Bioproject accession number PRJNA260750.

### Sporulation-Related Genes Profiles

An extensive literature search was performed in order to compile all spore- and sporulation-specific genes reported for the known spore-forming phyla (Actinobacteria, Cyanobacteria, Firmicutes, and Proteobacteria). Whether a protein encoded by those genes was submitted to UniProt or NCBI was then verified. These proteins, along with their homologs from other spore-forming species were downloaded and multiple sequence alignments were performed using Clustal Omega, with default parameters (Sievers et al., 2011). For these proteins, profiles were built using HMMER version 3.1 with default parameters (Eddy, 2011). An additional extended literature search was performed in order to catalog all reported spore-forming and non-spore-forming species of the four phyla (Actinobacteria, Cyanobacteria, Firmicutes, and Proteobacteria). The genomes of the reported spore-formers were downloaded from NCBI, when available. These genomes were thus screened using the generated profiles and the latter were updated. The updated profiles were used to screen the genome of *S. ureilytica* Lr5/4.

### Genome Analysis

#### Comparative Genomics

*Serratia ureilytica* Lr 4/5 full genome was compared to other genomes of *Serratia* species using the online comparative genomics tools EDGAR (Blom et al., 2009). A phylogenetic tree based on this comparison was generated. To construct a phylogenetic tree comprising several genomes (NCBI Accession numbers provided in Supplementary Figure 2) the core genes of these genomes were established. Alignments of the core genes were generated using MUSCLE (Edgar, 2004). Non-matching parts of the alignment were masked by GBLOCKS (Castresana, 2000) and subsequently removed. The remaining parts of the alignments were concatenated to one unique alignment used as input for PHYLIP (Felsenstein, 1993). This led to a phylogenetic tree, represented in Newick format.

#### Presence of Sporulation Genes

The genome of *S. ureilytica* Lr5/4 was screened for orthologs against the four above-mentioned datasets, using tBLASTn, under default parameters and an *E*-value cutoff of 1e^-11^. A tBLASTn run on the shuffled protein sequences as a negative control set showed no hit with an *E*-value lower than 4e^-4^. Each sporulation-related *S. ureilytica* Lr 4/5 gene was subjected to the following procedure:

##### Finding homologs

We searched for sequence-level similarities in UniProtKB using BLASTX on the UniProtKB website and default parameters. Then, using the best BLASTX hit, we considered (in this order): (1) links to orthology or phylogenomics databases; (2) links to databases of protein domain families. Links to orthology and phylogenomics databases were not available. Therefore, protein domain data was used instead. In this case, we selected the longest domain that matched our query, and downloaded Pfam (Finn et al., 2014) seed alignments for the domain. This resulted in sequences that were probably homologous to our query gene (since they contained the matching domain), but, in most cases, not necessarily *ortho*logous as a since paralog will contain the domain as well.

##### Building alignments

The query sequence was added to the Pfam seed alignment using Mafft (Katoh et al., 2002), using the option –keep-aligned and xyz so as to keep the original alignment intact and only aligning the query to it.

##### Computing and rooting trees

Using these alignments as inputs, maximum-likelihood phylogenetic trees were computed with PhyML (Le and Gascuel, 2008), using the XYZ model, maximum-likelihood estimates of amino acid and invariant frequencies, 12 substitution rate categories, and best topology search moves (-d aa -f a -v e -c 12 -a e -s BEST). In the general case, it was not possible to determine a suitable outgroup. This is because (i) in most cases the trees contain paralogs, and (ii) the phylogeny of bacteria is still unresolved, which entails that only a eukaryotic or archaeal homolog would be suitable – and these do not always exist. Therefore, we did not attempt to root the trees.

##### Closest relatives

Instead, we used the patristic distance (i.e., distance along tree branches) to infer the closest relative to the genes identified in *S. ureilytica* Lr 5/4. This differs from a simple genetic distance matrix in that the distances were actually estimated by maximum likelihood over a phylogeny and are therefore arguably more accurate. The distances were extracted with the Newick Utilities (Junier and Zdobnov, 2010).

##### Heat maps

The heat maps were produced with the heatmap() function from the R package.

## Results

### Isolation of *Serratia ureilytica* Lr5/4

The characterization of the strain Lr5/4 showed oval cells (0.5 × 1–3 μm) with a negative Gram-staining. Growth tests showed that Lr5/4 is a mesophile (temperature growth range between 10 and 37°C) with an optimum at 25°C. This range is not within the temperature measured at the sampling site (56°C), suggesting that the strain was present in the geothermal spring in a dormant state. Isolate Lr5/4 is also moderately halotolerant (growth up to 8% NaCl), able to develop over a vast pH range (from 3 to 11; optimal 5 to 6), and a facultative anaerobe, which are all characteristics that can be expected from a poly-extremophilic bacterium inhabiting the Chilean Altiplano. Based on the entire 16S rRNA gene sequence, isolate Lr5/4 belongs to the genus *Serratia*, with more than 97% identity to both *Serratia marcescens* and *Serratia ureilytica*. To establish the phylogenetic affiliation of the strain, a series of additional analysis were performed. While, MALDI-TOF characterization suggested that strain Lr5/4 belonged to *S. marcescens* (Supplementary Figure 1), PFLA analysis (Supplementary Table 1) showed that palmitic acid is the major fatty acid in the three strains. Based on the total fatty acid composition, Lr5/4 is more closely related to *S. ureilytica*, although the fatty acid profile of *S. ureilytica* type strain correlates better with *S. marcescens* than with Lr5/4. DNA/DNA hybridization analysis between Lr5/4 and its closest relatives (*S. ureilytica* DSM-16952 and *S. marcescens* DSM-30121) suggests that it is a member of *S. ureilytica*, with above 99% DNA/DNA relatedness to *S. ureilytica* DSM-16952. Nonetheless, DNA/DNA relatedness was also found to be above 70% with *S. marcescens* subsp. *marcescens*, which is the recommended threshold for delineating bacterial species (*18*). This observation is intriguing as it has been previously shown that DNA/DNA relatedness between the two reference species (*S. ureilytica* and *S. marcescens* subsp. *marcescens*) is only 43.7% (*19*). Finally, a phylogenetic tree constructed using all the available full genomes of *Serratia* isolates was constructed showing the relationship between the species that belong to this genus (Supplementary Figure 2). Considering all biochemical and molecular tests performed, we concluded that strain Lr5/4 represents a novel strain of *Serratia ureilytica.*

### Morphology of Dormant Cells in *S. ureilytica* Lr5/4

**Figure 2:**
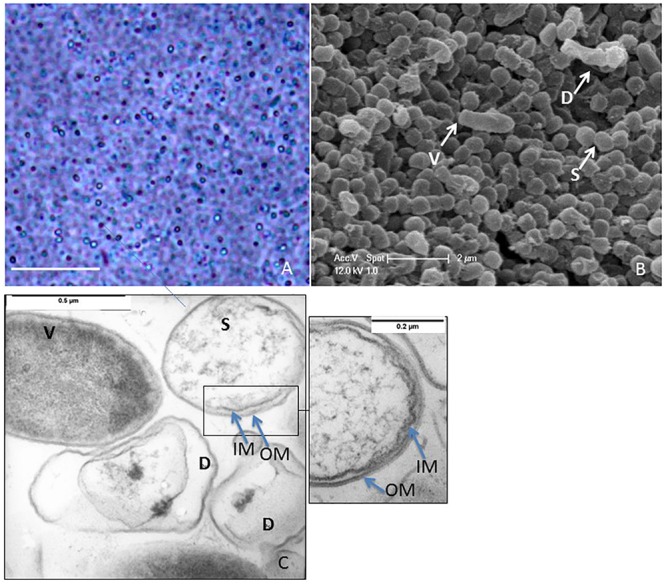
**(A)** Phase contrast microscopy of a 2-month old culture of Lr5/4. Phase bright objects of different sizes, are surrounded by lysed cells. Bar = 10 μm. **(B)** Scanning electron microscopy of vegetative (V), lysed (D), and dormant (S) cells. **(C)** Transmission electron microscopy (TEM) of vegetative (V), lysed (D), and dormant (S) cells. Noticeable black spots inside the lysed cells. Dormant cells are clearly shown to be double-membraned (see also inset).

After nutrient deprivation, *S. ureilytica* Lr5/4 differentiated into elliptic-spherical cell forms that appeared phase-bright at the phase contrast microscope (Figure 2A). The morphological characterization of dormant cells in strain Lr5/4 was further conducted using electron microscopy. Using scanning electron microscopy, vegetative cells display a bacillus-like shape of approximately 2.0 μm × 0.5 μm in size, while dormant cells corresponded to round spheres of 0.5 μm in diameter (Figure 2B). Using TEM and osmium (OsO_4_) fixation, the interior of vegetative cells appears stained while dormant cells present a clearer core surrounded by two thin outer layers (Figure 2C and inset). Although the exact composition of these two layers is not yet known, they prevent the penetration of the osmium tetraoxide (OsO_4_), used for fixation. The latter penetrated easily the cell wall and membrane of the vegetative form, which became dark gray (Figure 2C). OsO_4_ is used for fixation of spore structures of Firmicutes, Actinomycetes (Lechevalier et al., 1966), and *Myxococcus* (Müller et al., 2015). An increase in coat thickness hinders the penetration of OsO_4_, resulting in poor resolution of the inner coat and the spore protoplasm (Stevenson et al., 1972). The same has also been shown during germination experiments. In the latter, the protoplasm of spores is brighter and stains darker during germination as the spore coat is remodeled during outgrow (Hoeniger and Headley, 1968). Finally, experiments on the formation of resting light-refracting structures previously performed (Muliukin et al., 1997), have shown that TEM pictures of cells that resume their growth cycle stain dark, as observed also for *S. ureilytica* str. Lr5/4 (Figure 2C). This suggests that in spite of the apparent simplicity of the outer layers in the dormant form of strain Lr5/4, they are nonetheless impermeable to OsO_4_ and prevent staining. Overall the morphological characteristics of the dormancy cells in *Serratia* suggest that they differ in size and form to the vegetative cells.

### Dormancy Triggers

Although many environmental factors can hinder bacterial survival, nutrient deprivation is a well-studied dormancy triggering factor (Barton, 2005). Accordingly, *S. ureilytica* Lr5/4 dormant cells developed in response to starvation (after 3 months of growth on agar medium). Other environmental factors may also influence spore-formation, as well as resting cell production in non-spore-formers (Suzina et al., 2006), however, these factors have not been thoroughly tested in previous studies. Therefore, for this study, the roles of other known triggers of dormancy that could be relevant in the geothermal spring from which strain Lr5/4 was isolated (UV radiation, thermal shock, temperature extremes and desiccation) were also measured. Dormant cells of Lr5/4 were produced in response to UV radiation and thermal shock (Supplementary Table 2). In contrast, cells of Lr5/4 were largely tolerant to desiccation. Extreme temperatures (down to -80°C and up to 100°C applied overnight), did not induce dormancy in *S. ureilytica* Lr5/4. B. *subtilis* str. NEU1294 and *Anoxybacillus* sp. str. Lr10/3 responded to desiccation and extreme temperatures by producing endospores. On the contrary, *S. marcescens* DSMZ 30121, *S. ureilytica* DSMZ 16952, and *E. coli* str. NEU1006 did not produce any cyst-like cells. Although cyst-like resting structures have been previously reported for *E. coli* (Suzina et al., 2004), it appears that the specific environmental triggers that are characteristic of the Atacama Desert did not trigger cyst-production in the *E. coli* strain tested here. No spore or cyst production was observed in response to autoclaving. Autoclaving killed all cells tested.

### Resistance of Lr5/4 Dormant Cell

In order to evaluate the resistance of the dormant forms produced by *S. ureilytica* Lr5/4, cyst-like cells from 90-day old and heat-shocked cultures were challenged with multiple environmental stressors. Dormant forms of Lr5/4 were able to withstand and generate an actively growing culture after wet heat-shock at 75°C, 240 min UV radiation exposure, and incubation at extreme temperatures. This was comparable to the survival of endospores from the endospore-forming bacilli *B. subtilis* and *Anoxybacillus* sp. Lr10/3, although the latter resisted to 120 min UV radiation exposure (Supplementary Table 3). However, cells of *S. marcescens* DSMZ 30121 and *S. ureilytica* DSMZ 16952, as well as cells of *E. coli* did not survive these treatments. Autoclaving killed all spores and cells tested. When dormant forms of Lr5/4, *B. subtilis* and *Anoxybacillus* sp. Lr10/3 were subjected to multiple stressors applied simultaneously (starvation, desiccation, and UV radiation), only the endospores of the Firmicutes species could be revived, while Lr5/4 did not survive.

Although for most of the stressors studied, the precise molecular mechanism involved in resistance is unknown, in endospores of Firmicutes the protection of DNA in response to wet heat is conferred by the accumulation of dipicolinic acid (DPA) in the core (Setlow et al., 2006). The presence of DPA (Brandes Ammann et al., 2011) in the dormant cells of Lr5/4 was assessed on dilutions of a 3-month old culture (Supplementary Figure 3). The DPA content per dormant cell was calculated to be between 2.36 and 2.55 × 10^-7^ μg/dormant cell, a value nearly 14 times lower than the DPA concentration in spores of *B. subtilis*. DPA was also reported as detectable in the case of *S. marcescens* subsp. *sakuensis* [10 mg (g bacteria)^-1^] (Ajithkumar et al., 2003), but the values cannot be compared to those obtained here as the dormant to vegetative cell ratio was not provided in the case of *S. marcescens* subsp. *sakuensis*.

### Genomic Imprints of Dormancy in Lr5/4

**Figure 3:**
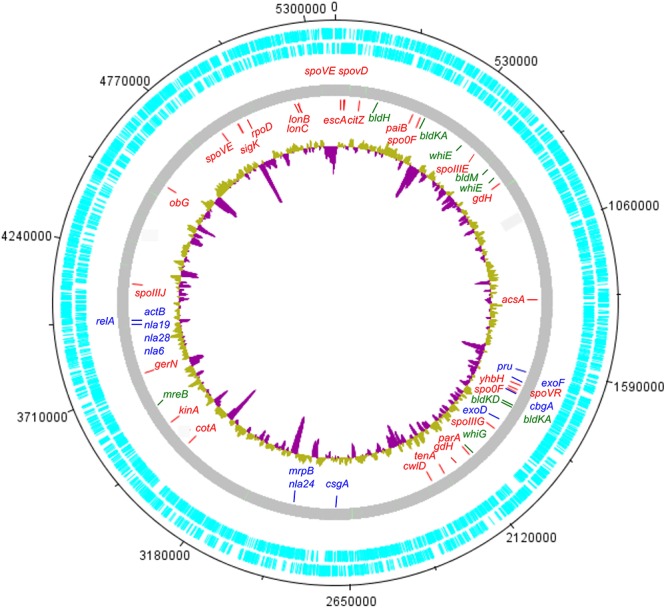
Circular representation of the *S. ureilytica* Lr5/4 genome. Genes related to sporulation are spread throughout the chromosome. Genes indicated in red show homology to genes related to endospore-formation in Firmicutes, in blue to myxospore-formation in delta-Proteobacteria and in green to exospores in Actinobacteria. The inner circle in yellow (A+T%) and purple (G+C%) represents the G+C% content variation throughout the bacterial chromosome.

**Figure 4:**
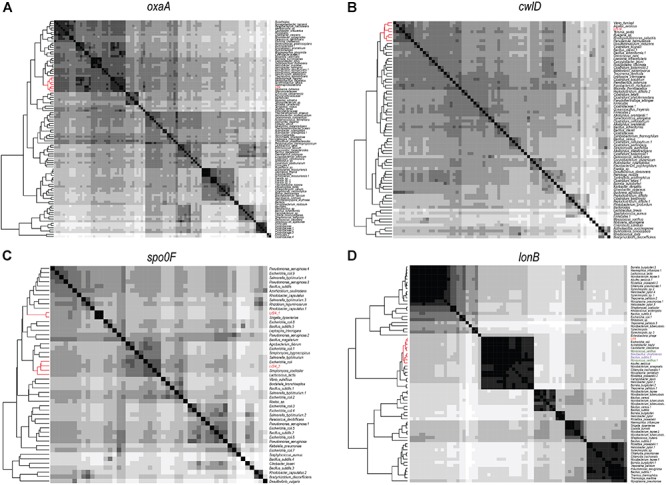
Heatmaps showing the phylogenomic neighborhood for four genes related to sporulation found in the genome of *S. ureilytica* Lr5/4 (highlighted in red). In panels **(A–C)**, the highest similarity for the sporulation genes lies with phylogenetically closely related taxa (indicated in red). This was true for 25 genes. In panel **(D)**, the only exception is shown. Homologs of the *lonB* gene are more similar between species from distant phyla, than from species of the same phylum. Strain Lr5/4 has a *lonB* gene that is found to bear closer similarity to its homolog in *Myxococcus* (green) and *Bacillus* (purple), rather than to homolog genes in γ-Proteobacteria.

In order to investigate the genomic imprints of dormancy in strain Lr5/4, the complete genome of the strain was sequenced (5.39 Mb in size, 59.2% G+C) and annotated. We next compared the 5,056 annotated genes to genes reported to be involved in sporulation in other clades. The molecular pathways for bacterial cellular differentiation to the formation of a spore are well described in Actinobacteria, Cyanobacteria, Firmicutes, and Proteobacteria. Therefore, a database with all the known genes involved in dormancy in these taxa (Supplementary Table 4) was built and the genome of *S. ureilytica* Lr5/4 was screened for the presence of those genes. The detection of these genes using a BLAST search is indicative rather than demonstrative of the presence of this genetic determinants and further experiments, including gene expression and functional characterization during sporulation, need to be performed. Therefore, these results should be regarded with caution. Nonetheless, significant homology was detected between 12 Lr5/4 loci and myxospore-formation genes, nine in actinobacterial exospores, and 24 genes involved in spore formation in the Firmicutes (Supplementary Table 5). These genes participate in peptidoglycan synthesis, signaling, and transcription regulation. These genes are dispersed throughout the genome of Lr5/4 (Figure 3). The phylogenetic analysis of the genes that are potentially involved in dormancy show that most of them are likely of proteobacterial origin (Figure 4), suggesting they have not recently been acquired via horizontal gene transfer. A thorough phylogenomic and functional analysis of the dormancy-related genes showed that they contain conserved domains homologous to proteins that, in other species, participate mainly to general stages of cell division and sensing (Table 1), with homologs reported also in non-dormant bacteria.

**Table 1:** Presence of the 26 genes detected in the genome of *Serratia* in multiple phyla.

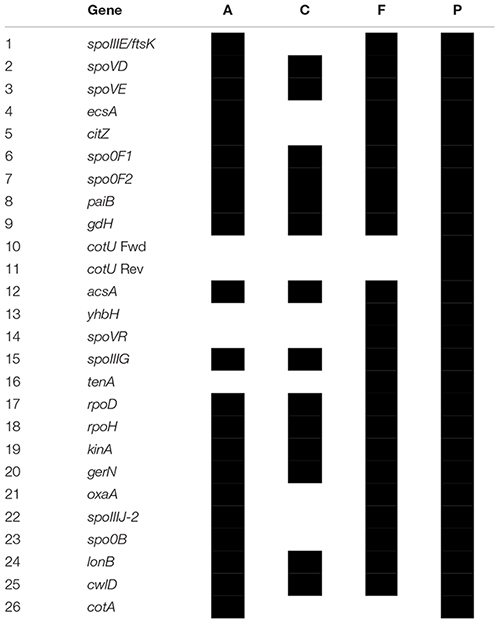

*A, actinomycetes; C, cyanobacteria; F, firmicutes; P, proteobacteria*.

## Discussion

In this work we report the formation of cyst-like cells in a novel *Serratia* strain. We show for the first time that *S. ureilytica* Lr5/4 produces a differentiated cell form that is more resistant than vegetative cells. This is probably due to its denser cellular structure. Furthermore, to date, only the vegetative form has been observed to divide under optimal conditions. When the differentiated cell types are cultured under optimal conditions, only the vegetative cell forms are observed under the microscope. In dormant state, cells contain dipicolinic acid, a compound that protects the genetic material from degradation upon exposure to wet heat (Setlow et al., 2006). For all these reasons, we argue that the differentiated cell type of *Serratia* Lr5/4 is a resting resistant cellular structure. Our results show that cellular differentiation occurs under starvation, strong temperature fluctuations (shocks) and UV radiation. These parameters characterize the environment of the Chilean Altiplano from which this strain was isolated. Our experiments lead us to the conclusion that the formation of such structures is a likely adaptation to environmental extremes.

Finding cyst-like cells in *S. ureilytica* Lr5/4 provides another example in the extending list of resistant cyst-like-producing, non-spore-forming bacteria. This list includes *Pseudomonas aeruginosa* (Zechman and Casida, 1982), *Micrococcus luteus* (Mulyukin et al., 1996), *Thioalkalivibrio versutus* (Loiko et al., 2003), *Sinorhizobium meliloti* (Loiko et al., 2011), and *Arthrobacter globiformis* (Demkina et al., 2000), among others (Suzina et al., 2004). Dormancy seems now to be extended to almost every known bacterial phylum, and it has been even suggested that a common “sporulating” ancestor to bacteria might have existed in the past (Tocheva et al., 2016). Indeed, the ability to enter into a dormant and resistant cellular state would have provided a clear advantage to bacteria in an early Earth exposed to frequent meteoritic bombardments and alternating periods of boiling and freezing temperatures (Nicholson et al., 2002; Müller et al., 2014). Although beneficial under fluctuating conditions, untimely dormancy has severe consequences and would be under negative selection under stable conditions (Maughan et al., 2009). Therefore, selective pressure from the environment seems to be an essential condition for this mechanism to be maintained and deployed. In the case of the Chilean Altiplano, poly-extreme conditions could have led *S. ureilytica* Lr5/4 to evolve or to express this trait. The same could be the case for Archaea, in which the formation of spherical forms in response to a decrease in water activity (Fendrihan et al., 2012), appears to correlate with the survival and re-culturing of halophilic Archaea from salt deposits as old as the Permian (Stan-Lotter et al., 2002; Gruber et al., 2004). In environments favoring dormancy (such as polyextremophilic environments), this survival strategy could thus be found among bacterial and archaeal groups previously considered non-dormant. In contrast, in environments in which dormancy incurs a fitness cost, this trait could have been lost explaining the diversity of non-dormant groups reported nowadays.

To date, harmful conditions [extreme or autolyzing (Muliukin et al., 1997)] seem to trigger the formation of resting cells in various microorganisms. However, whether the molecular mechanism for the formation of resting structures is similar among bacterial taxa remains to be shown. It has been hypothesized that cellular differentiation in these non-spore-forming organisms follow an alternative molecular pathway to the pathways described in the known spore-forming phyla (Suzina et al., 2004). The genetic analysis of the present study shed light on this unknown molecular mechanism. The results of the comparative analysis of the genome of *S. ureilytica* Lr5/4 with a database of genes involved in spore formation are striking. With the exception of Cyanobacteria, similarities were found. The homologous sporulation genes found in *S. ureilytica* Lr5/4 were either transcriptional factors, such as *sigG, sigK*, or *bldH*, or are homologous to house-keeping genes related to cellular division, such as *spoIIIE*, *mreB*, *exoD*, or *exoF*. These observations may lead to the suggestion that specialized transcriptional factors may be used for the alternative transcription of house-keeping genes for cellular differentiation, a rather economic alternative to the maintenance of a costly spore-formation machinery. The existence of *spoVR* homologs in different organisms appears as an important commonality to spore production. The SpoVR protein is responsible for spore cortex assembly in Firmicutes, but it is also found in myxospore-formation. The spore peptidoglycan in Firmicutes and Myxococcales differs from the one in vegetative cells (Atrih and Foster, 1999; Bui et al., 2009). Therefore, the presence of this gene in a resting cyst producer, like *S. ureilytica* Lr5/4 also highlights the need for the presence of specialized genes in the molecular pathway of cyst-like production.

An extensive genetic analysis can only provide candidates to the molecular pathways. Whether these genes are indeed expressed while the bacterial cells are stressed, should be investigated for all cyst-producing organisms using gene expression analysis. A phylogenomic analysis of those genes, however, answers two other fundamental questions: what is the origin of dormancy in strain Lr5/4? And, do these genes correspond to a “dormancy gene core” widespread among unrelated taxa? Two scenarios have been proposed for the presence of dormancy in *Serratia*. The first scenario proposes dormancy is the result of gene transfer across unrelated bacterial genera (Ajithkumar et al., 2003), a phenomenon that has been reported as commonly occurring among hyperthermophiles in geothermal sites (van Wolferen et al., 2013). This scenario could be possible because genes related to sporulation have been found in genomes of non-sporulating species (Onyenwoke et al., 2004). However, in our opinion, gene transfer is unlikely because the formation of a fully functional spore is an exquisitely complex process that in the case of Firmicutes (the most studied model in terms of genetics and the mechanism of sporulation), requires a minimum of 60 genes, located throughout the bacterial chromosome and acting co-ordinately at different stages (Galperin et al., 2012). The second scenario is that dormancy is an ancient train within this genus, expressed only under particular conditions. The phylogenetic analysis of genes related to dormancy that are found in the genome of *S. ureilytica* Lr5/4 shows that the phylogenetic neighborhood of these genes is clearly proteobacterial, providing proof of the ancestry of these genes within the clade of Proteobacteria. Our phylogenomic analysis also revealed that these genes are not uniquely found in the genome of *S. ureilytica* Lr5/4, nor only acquitted among Proteobacteria. On the contrary, these genes are widespread among various sporulating taxa. These genes are required for cellular division, or control at crucial checkpoints. A previous analysis on Firmicutes sporulation-related genes showed that some of these genes may also be found in non-spore formers (Galperin, 2013). These genes are also shown to be involved in cell division and signaling/sensing and can be remnants of a sporulating ancestor (Onyenwoke et al., 2004). With the exception of *cotU*, which was solely found in Proteobacteria, the other genes detected in the genome of *S. ureilytica* Lr5/4 are found in two or more sporulating phyla. This observation suggests that sporulating organisms might share common dormancy-related genes. However, although homologs, whether these genes play a role in sporulation or have a function related to dormancy in all phyla needs to be verified in future studies.

## Conclusion

The widespread phylogenomic distribution of dormancy genes should be seen under the prism of widespread cellular differentiation in bacteria, promoted under harsh conditions. The output of the phylogenomic and functional analysis of dormancy genes highlights that cellular differentiation to a more resistant structure, for instance, is highly related to cellular division. Moreover, it also shows that the choice between division and differentiation depends on cellular sensing. Since many non-spore-formers produce resting structures (Muliukin et al., 1997; Suzina et al., 2004, 2006), the molecular mechanism for such differentiation capability may not involve a complex specialized pathway. As our findings indicate, the production of resistant resting cells from previously known non-spore-forming bacteria, may involve specific cell-division genes. An important step into the understanding of dormancy in *S. ureilytica* Lr5/4 is to discover all the genes involved in cell differentiation and to reconstruct the molecular pathway, but also study the levels of expression for each gene. Future work should involve an expression analysis under the two morphotypes (vegetative and resting) for all cyst-producing bacteria. However, with this work we support the scenario that dimorphic bacterial cells are a widespread phenomenon, showing that one of the two cell morphologies, the most resistant, is produced after exposure to harsh environmental conditions. The ecological significance of this mechanism is high, since survival seems to be feasible to an increasing number of extreme conditions, but also it seems to be extended to more and more bacterial phyla.

## Author Contributions

SF performed physiological and bioinformatics analysis and identification and wrote the manuscript. TJ performed bioinformatics analysis. TW participated to the sampling and performed isolation. WK, IP, and AA-D helped for the collection of physiological data. VM participated to the sampling. RL performed the MALDI-TOF analysis. JS performed the lipid analysis. SJ performed the automated genome annotation. PC supervised the automatic genome annotation. CD coordinated access to the sampling site and participated to the *in situ* collection of data and sampling. PJ designed the study, participated to the data analysis, and wrote the manuscript. All authors contributed and approved the final version of the manuscript.

## Conflict of Interest Statement

The authors declare that the research was conducted in the absence of any commercial or financial relationships that could be construed as a potential conflict of interest.
