# Supplementary material for: Adaptive Strategies in a Poly-Extreme Environment: Differentiation of Vegetative Cells in Serratia ureilytica and Resistance to Extreme Conditions
Source: Front Microbiol. 2019 Feb 5;10:102. doi: 10.3389/fmicb.2019.00102 (PMC6370625; doi:10.3389/fmicb.2019.00102)
Supplement: Supplementary file 2 [file Image_1.pdf]

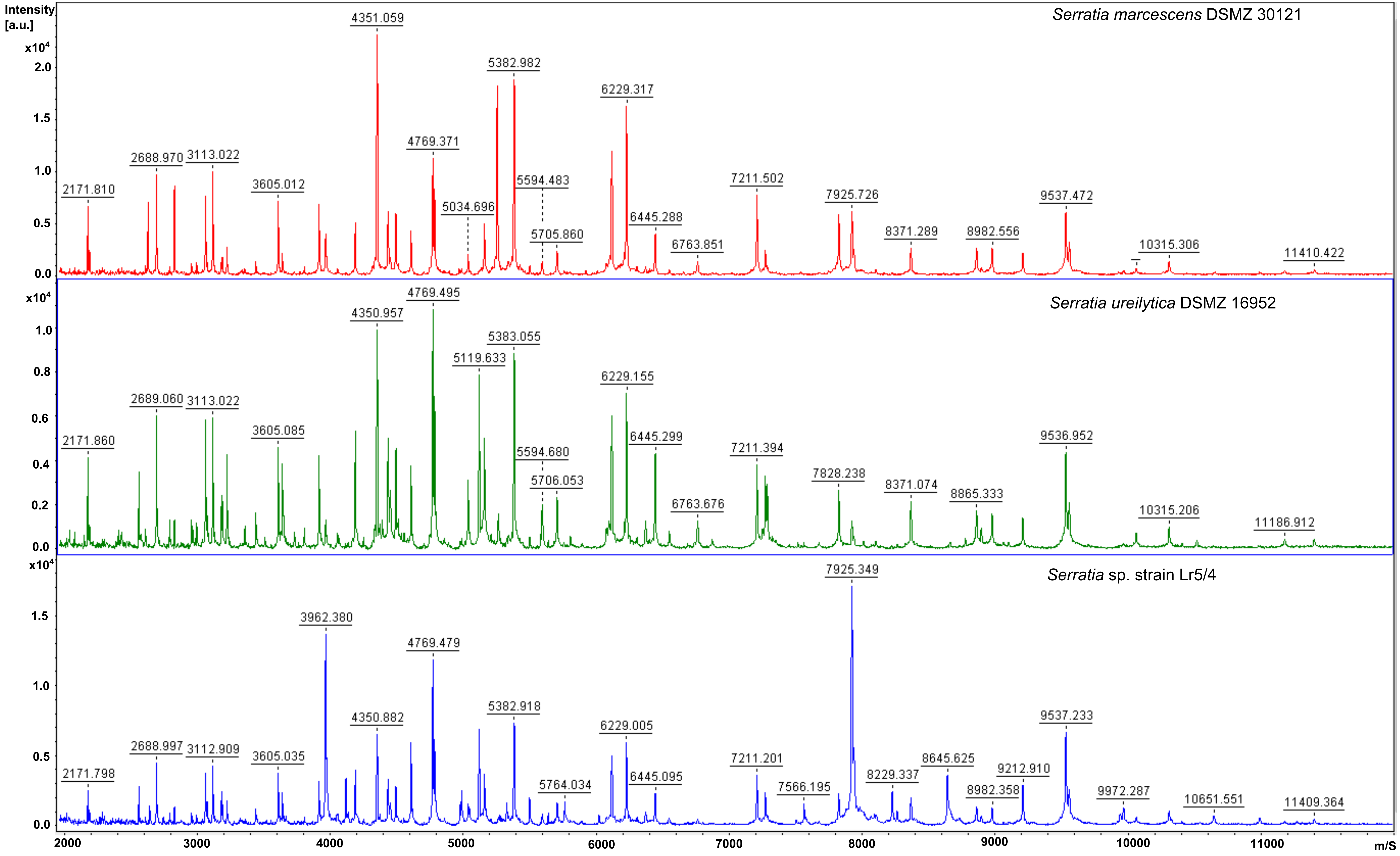

Supplementary Figure 1. Comparison of matrix-assisted laser desorption/ionization-time of flying (MALDI-TOF) profiles of strain Lr5/4, *S. marcescens* subsp. *marcescens* and *S. ureilytica*.
