# Supplementary material for: Adaptive Strategies in a Poly-Extreme Environment: Differentiation of Vegetative Cells in Serratia ureilytica and Resistance to Extreme Conditions
Source: Front Microbiol. 2019 Feb 5;10:102. doi: 10.3389/fmicb.2019.00102 (PMC6370625; doi:10.3389/fmicb.2019.00102)
Supplement: Supplementary file 3 [file Image_2.pdf]

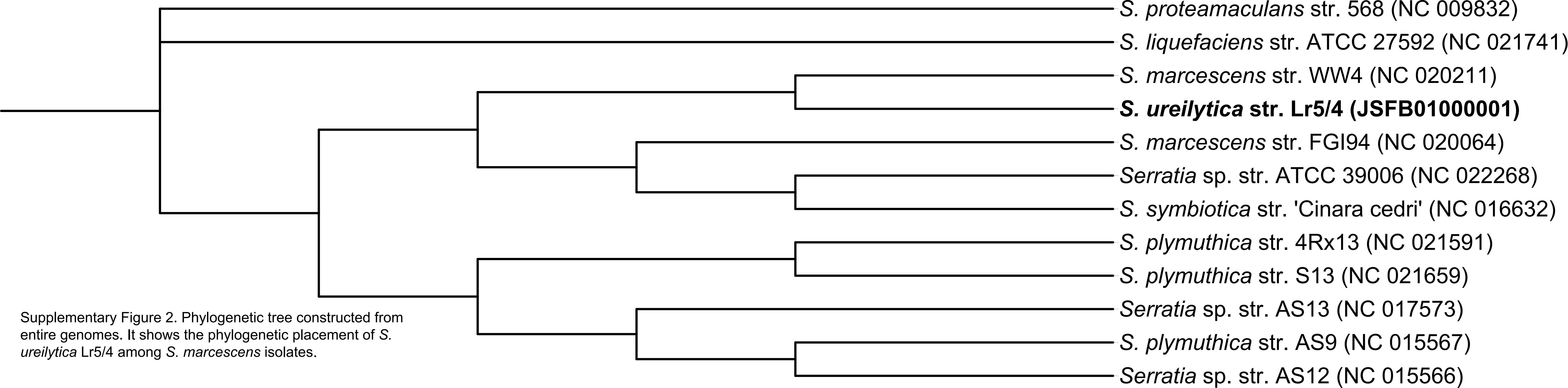

Supplementary Figure 2. Phylogenetic tree constructed from entire genomes. It shows the phylogenetic placement of *S. ureilytica* Lr5/4 among *S. marcescens* isolates.
