# Supplementary material for: Adaptive Strategies in a Poly-Extreme Environment: Differentiation of Vegetative Cells in Serratia ureilytica and Resistance to Extreme Conditions
Source: Front Microbiol. 2019 Feb 5;10:102. doi: 10.3389/fmicb.2019.00102 (PMC6370625; doi:10.3389/fmicb.2019.00102)
Supplement: Supplementary file 4 [file Image_3.pdf]

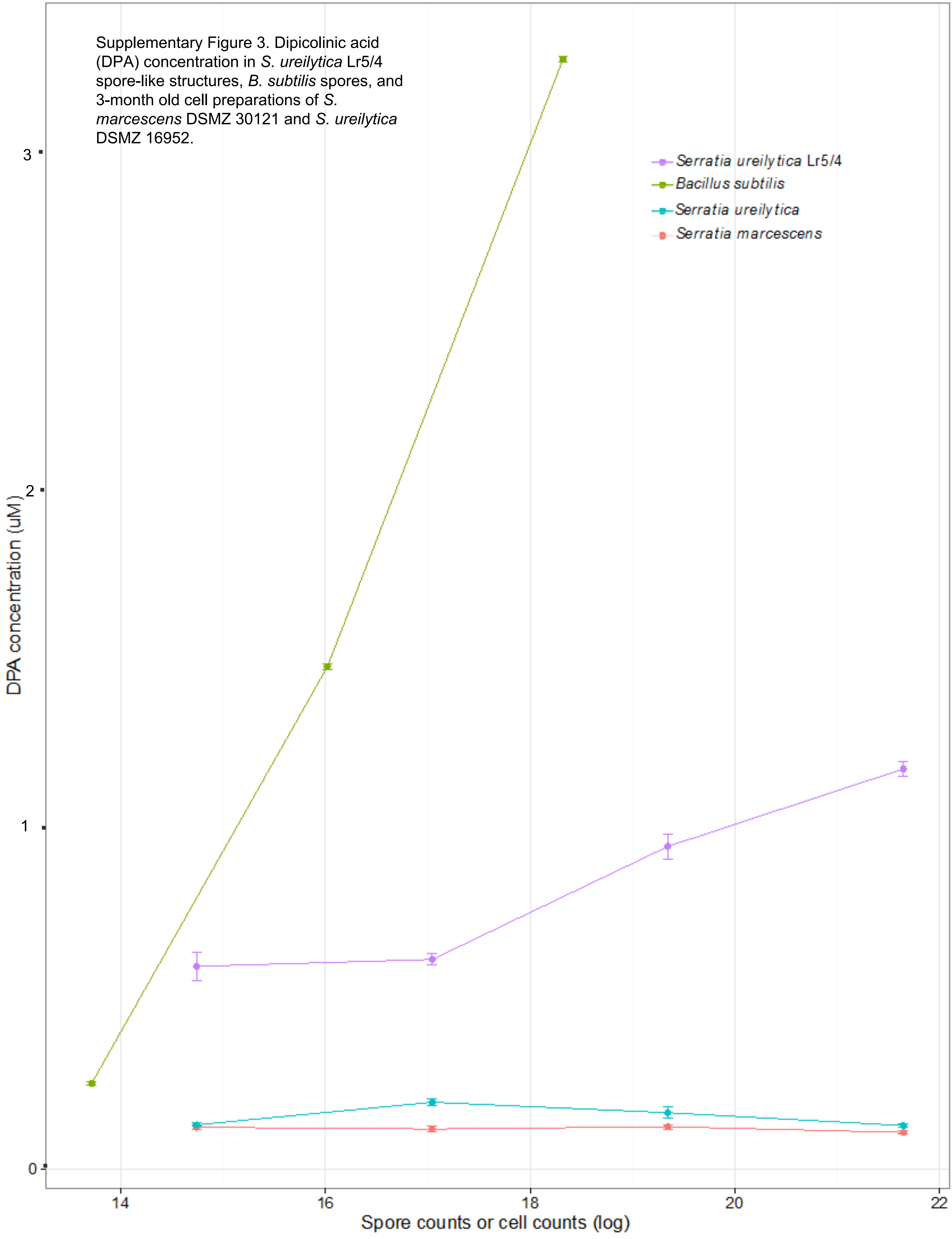

Supplementary Figure 3. Dipicolinic acid (DPA) concentration in *S. ureilytica* Lr5/4 spore-like structures, *B. subtilis* spores, and 3-month old cell preparations of *S. marcescens* DSMZ 30121 and *S. ureilytica* DSMZ 16952.
